# Analysing Patterns in Electronic Optometry Referrals: Feasibility and Methodology

**DOI:** 10.1007/s44402-026-00019-1

**Published:** 2026-03-06

**Authors:** Abdullah F. Alotaibi, Matthew Jinkinson, Ketan R. Parmar, Glen P. Martin, Philip B. Morgan, Robert A. Harper

**Affiliations:** 1 Division of Pharmacy and Optometry, The University of Manchester, Manchester, UK; 2 Greater Manchester Eye Health Network, NHS Greater Manchester Integrated Care, Manchester, UK; 3 Division of Neuroscience and Experimental Psychology, School of Biological Sciences, The University of Manchester, Manchester, UK; 4 Centre for Health Informatics, Division of Informatics, Imaging and Data Sciences, The University of Manchester, Manchester, UK; 5 Eurolens Research, Division of Pharmacy and Optometry, The University of Manchester, Manchester, UK; 6 Manchester Royal Eye Hospital and Manchester Academic Health Sciences Centre, Manchester University NHS Foundation Trust Manchester, Manchester, UK

**Keywords:** Electronic referrals, Feasibility study, Health inequality, Optometry referrals, Primary eyecare, Referral patterns, Socioeconomic deprivation

## Abstract

**Purpose:**

There may be considerable untapped potential in using large-scale electronic primary care optometry referral data to understand referral patterns. This study aims to evaluate the feasibility of using different analytical methods for this type of dataset.

**Methods:**

A total of 12,339 electronic referrals made by primary care optometrists in November 2023 were examined. The dataset contained 36 demographic and clinical variables. Preprocessing involved categorising and normalising referral conditions and merging similar attributes to enhance consistency. The feasibility of descriptive evaluations of referral conditions to both ophthalmology in secondary care and within primary care was explored, and a regression analysis was conducted to investigate potential associations between patient sex and referral patterns. A spatial analysis was also conducted to map the geographic distribution of referrals and their association with social deprivation.

**Results:**

Of all referrals, 77.3% were directed to ophthalmology in secondary care, 14.1% within primary eyecare optometry and non-optometric services, with the remaining 8.5% being unspecified. Cataracts (17.2%), glaucoma (13.5%) and YAG laser capsulotomy (12.1%) emerged as the most frequent referral reasons. Regression analysis identified significant sex differences, where referral proportions for female patients were higher than males for conditions such as glaucoma and neuro-ophthalmology (*p* = 0.001). Spatial analysis revealed no significant difference between the referral ratio and the deprivation level.

**Conclusions:**

This study demonstrated viability for evaluating electronic optometry referrals. Despite data limitations, these findings indicate that data quality and scope can support established analytical methods. Moreover, the scale of data variables suggests expanding sample sizes and extending time windows could reveal clinically informative patterns in referrals. Future investigations may further validate findings, helping to understand local, regional or national referral patterns within optometry and potentially address inequalities in eyecare.

Key Points

- This feasibility study demonstrated that large-scale evaluation of electronic optometry referrals is achievable through the integration of multiple routine datasets.
- A structured process enabled the categorisation and standardisation of referral types, with cataract, YAG laser capsulotomy and glaucoma accounting for the majority.
- The analysis methods can be used to evaluate data from a larger geographical area and/or longer time period, offering opportunities for targeted service planning and quality improvement.

## Introduction

One fundamental role of optometric care globally is referring patients to other clinicians—such as ophthalmologists or general medical practitioners (GPs)—for further investigation, formal diagnosis or management. The precise referral process is dependent on the structure of local healthcare systems and the position of optometrists within these systems [1].

Evaluating referral data in general healthcare systems is a widely used method for evaluation of a range of healthcare services, including optometry [2]. Examples of studies that have examined referrals by optometrists typically focus on single conditions—for instance, age-related macular degeneration (AMD) [3, 4], cataract [5, 6] and glaucoma [7, 8]. Other studies evaluated referral decisions using clinical vignettes to explore the relationship between referral patterns and optometrists’ post-qualification experience [9]. Referral decision-making in optometry has also been assessed using Bayesian learning approaches [10]. However, a comprehensive overall evaluation of optometric referrals, taking into account multiple aspects and using large-scale data, has not been attempted previously [11]. Therefore, there is a lack of a comprehensive overview of the current state of optometry-initiated referrals in the UK, despite there being over 1.1 million referrals made from primary eye care to ophthalmology in secondary care services in the UK in 2023 [12, 13].

Over the past three decades, the referral process for primary care optometrists in the UK has evolved significantly. Optometrists first gained the right to refer patients directly to ophthalmology in secondary care following the introduction of an amended Opticians Act in 1989. This right was further expanded in 1994 to include referrals to other eye care providers. The NHS Primary Care Act of 1997 and GOC’s rules on referral subsequently allowed optometrists to refer patients based on their clinical judgement, and in 2005, the GOC permitted optometrists to refer patients to more experienced colleagues [14].

The National Health Service (NHS) first introduced an electronic referral system, Choose and Book, for GPs in 2005–06, which was replaced in 2015 by the NHS e-Referral Service (eRS). Initially limited to GP referrals into ophthalmology secondary care, eRS was intended to expand to other referrers, including primary care optometry. However, progress has been slow due to technical, financial and governance barriers [15]. Early access required costly NHS network connections and SmartCard authentication, while integration with optical practice management systems demanded significant supplier input. Adoption was further constrained by limited IT support, digital safety compliance and training and funding challenges, particularly in under-resourced regions.

To address these issues, NHS England commissioned the Electronic Eyecare Referral System (EeRS), a secure digital platform enabling two-way exchange of referral data, including images. Identified as a key deliverable in the 2021–22 NHS planning guidance, EeRS aims to modernise referrals and reduce avoidable outpatient appointments [16]. Yet, uptake has varied across England, with some areas continuing to rely on paper or alternative systems and referral practices also differ across the other UK nations [17].

No published evaluation has determined whether electronic referrals consistently enhance the accuracy or quality of referrals [18, 19]. Nevertheless, the digital nature of the NHS EeRS system presents unique opportunities for analysing a high volume of referrals, potentially offering analyses and insights previously very difficult to obtain, including, for example: referral patterns for ocular conditions; patient demographics; the impact of additional qualifications or other practitioner variables on referral quality and quantity; adherence to national guidelines and the impact of enhanced services in England. Patient demographic information is generally of moderate to high quality because of the mandatory requirement to collect it within e-referral systems [20]. These data could offer further insight into links between patient demographics and particular conditions or reasons for referral, which could inform future investigations into the eyecare pathways. Spatial analysis (evaluation of data in a geographical context) of eye referrals and conditions has not been studied extensively. An example of linking geographical data is through the use of Lower Layer Super Output Areas (LSOAs). LSOAs represent geographical groupings of approximately 1500 individuals, and can be linked to additional datasets to provide deeper insights [21]. Spatial analysis has previously been applied to other referral types, such as emergency pituitary referrals [22], psychiatry referrals [23] and outpatient respiratory referrals [24], but not to optometric referrals. Applying spatial analysis to optometry referrals could uncover social and environmental factors influencing condition rates [25]. Spatial analysis may offer a comprehensive understanding of the distribution of referred conditions and highlight geographical disparities [25].

As the first known study to examine large-scale data from electronic primary care optometry referrals, this project included a feasibility assessment of the overall approach. This study had three aims. Aim 1 was to summarise the clinical and demographic characteristics of the referred sample, data quality (e.g., level of missing data), the stated referral urgency and putative diagnoses. Aim 2 was to test the feasibility of investigating associations between patients’ demographic details (e.g., age and sex) and referral patterns to ophthalmology in secondary healthcare, including conditions such as glaucoma, cataract and other referral reasons. Aim 3 was to explore the feasibility for creating a map of optometry referrals.

## Methodology

### Data Acquisition

The dataset, obtained from NHS England North West (EeRS), comprised 12,339 anonymised electronic optometry referrals from the Northwest of England, covering data for November 2023. The dataset was received as a Microsoft Excel file (Microsoft.com) containing the information detailed in Table 1.

**Table 1:** Data fields received within the raw referral dataset.

Data fields title	Data field description	Data type
Age at activity	Age	Numeric
Postcode usual	Part of postcode, use only to link with (LSOAs)	Text
Stated gender	Sex	Numeric
Service type	Eye condition types	Text
Referral pathway l2	Destination of the referrals	Text
Referral type	Eye condition types	Text
Priority type	Priority of referral: routine, urgent and emergency	Text
Clinic name	Clinic that received the referrals	Text
Suggested Options	Indicates which NHS Clinical Commissioning Groups that the patient’s GP is associated with.	Text
Suggested not optional	Indicates which platform was used to send the referral	Text

Each field is accompanied by a description and the corresponding data type (e.g., numeric, text), providing an overview of the structure and content of the dataset used for analysis. “Referral pathway l2” denotes the destination of the referral within the ophthalmic care pathway.

*GP* general medical practitioner, *LSOA* lower layer super output area, *NHS* National Health Service.

### Evaluation Procedure

#### Data Cleaning and Organisation

First, the dataset contained multiple columns with different titles that provided the same information as other columns. For instance, there were three columns indicating the reason for referral, all of which contained similar data. In addition, two columns were found that showed whether the referral was to ophthalmology in secondary care or primary eye care. No columns were deleted or edited; instead, Python 3 (Python Software Foundation, python.org), run within Google Colab (Google LLC, colab.research.google.com), was used for the analysis, and only one of these columns was selected for the analysis process. Tabular data were managed with pandas, numerical operations with NumPy, and figures were produced using matplotlib and seaborn, with additional custom graphical elements created using the matplotlib.patches and matplotlib.lines modules. Spatial analyses and choropleth maps of referrals by LSOAs were generated using QGIS 3.40.5 (QGIS Development Team, qgis.org).

Part of the postcodes and their corresponding identifiers were extracted into a separate spreadsheet. These were converted subsequently to geographic coordinates (longitude and latitude) using Google Sheets extensions (Geocode for Sheets and Mapping Sheets workspace.google.com/marketplace/search/geocode). A verification process was then performed by manually selecting various postcodes and confirming that identical postcodes corresponded to the same coordinates. The coordinate data were then appended to the original dataset.

Each postcode was linked to its respective LSOAs in order to give an overview of a larger area, and to link this with the Index of Multiple Deprivation (IMD). The LSOAs data were sourced from the UK Government’s GeoPortal (geoportal.statistics.gov.uk), utilising the ‘Postcode to 2021 Census Output Area to LSOAs 2021’ dataset, updated on 12 March 2024. IMD data was obtained from the Office for National Statistics (ons.gov.uk/). IMD scores range from 1 (most deprived) to 10 (least deprived). These scores were assigned to each LSOA within the dataset, and referral ratios were calculated subsequently for each deprivation decile, enabling a direct comparison of referral patterns across different levels of deprivation.

#### Categorisation of Referral Types

The referral reasons within the dataset were categorised into three groups: “Referrals to Ophthalmology in Secondary care”, “Referrals to Primary Eyecare” and “Not Otherwise Specified”. The referral pathway column showed whether referrals were directed to primary eyecare or ophthalmology in secondary care, while the referral type column indicated the reason for referral, including those listed as ‘Not Specified’ or ‘Not Otherwise Specified’. This categorisation facilitated comparative analyses between the groups based on the ultimate target of the referral.

- Referrals to Primary Eyecare: Referrals sent to other community optometrists or GPs.
- Referrals to Ophthalmology in Secondary care: Referrals directed to secondary care ophthalmology.

Not Otherwise Specified: Referrals sent to ophthalmologists without a specified reason. These referrals were to ophthalmology in secondary care, but where the clinic type is for general ophthalmology, rather than specific to the condition found requiring referral.

To streamline the categorisation, similar referral types with different names but shared characteristics were consolidated. For instance, referrals to “squint / ocular motility” and “orthoptics” were grouped under “Adult Binocular Vision Referral” due to their similar nature.

### Data Analyses Aligned with Research Aims

#### Aim 1: Descriptive Analysis

Following the integration of multiple datasets, the dataset was examined for missing findings and assessed for internal consistency across variables. As part of the data preparation process, the extent of missing entries was reviewed, and consistency in key variables was verified. Upon confirming the completeness and integrity of the dataset, descriptive analyses of referral categories and types were conducted, with the results presented using a range of graphical outputs.

#### Aim 2: Regression Analysis

The dataset was divided into “Referrals to Ophthalmology in Secondary care” and “Referrals to Primary Eyecare”. Logistic regression analysis was performed to examine the relationship between sex and the conditions associated with each referral type. To control for Type I errors arising from multiple comparisons, the Bonferroni correction was applied [26]. Additionally, Chi-squared tests were conducted to calculate *p*-values for the association between sex and each referral reason. The age distribution of referred patients was also examined, and graphical summaries were generated to illustrate the variation across selected conditions.

#### Aim 3: Visualisation Analysis

A spatial mapping approach was undertaken to examine and visualise the dataset. The dataset encompassed the 36 former NHS Clinical Commissioning Groups (CCGs) in Northwest England, with most referrals processed via the NHS eRS. Approximately 3000 distinct areas were generated from postcodes linked to LSOAs to generate a visualisation map of referrals. A ratio-based approach was used to explore the distribution of referral types across different local areas. First, all service-type labels were standardised (e.g., adjusting for inconsistencies in wording, punctuation or spacing) to consolidate similar referral types under consistent category names. After these categories were established, the dataset was grouped by the defined local area codes and referral types to produce counts of each referral type.

For each local area, the total number of referrals was calculated. The proportion of each referral type in a given area—termed the referral ratio—was determined by dividing the individual referral-type count by the overall referral count in that area. Finally, the data were converted into a wide-format table with local areas as rows and referral types as columns. This process produced a clear overview of how referrals are distributed across referral categories within each locality. The resulting dataset was saved in a suitable file format for subsequent analyses and visualisations.

#### Ethical Considerations

This study evaluated pre-existing referral data, specifically routine NHS data, without intervention. The data was provided by the North West Electronic Eyecare Referral System (EeRS) Services Project Team to assess its potential for further analyses. The use of partial postcode data to explore associations with other datasets, such as LSOAs and IMD, are not deemed identifier information. According to The University of Manchester’s Ethical Research guidance (training.itservices.manchester.ac.uk/uom/ERM/ethics_decision_tool/story.html), this project was classified as “service evaluation and improvement initiative” and therefore did not require ethical approval.

## Results

The findings are organised in alignment with the three research aims. The dataset was received in a complete state, with no missing data or empty cells in the Excel file. The dataset contained 12,339 optometry referrals, with patient ages ranging from 0 to 104 years and a mean age of 61.4 ± 22.3 years.

### Descriptive Overview (Aim 1)

The distribution of referrals across the categories revealed that “Referrals to Ophthalmology in Secondary care” comprised the majority, accounting for 9543 (77.3%) referrals, followed by “Referrals to Primary Eyecare”, which constituted 1744 (14.1%) referrals, while “Not Otherwise Specified” referrals totalled 1052 (8.5%). Table 2 shows the descriptive analysis of the dataset.

**Table 2:** Characteristics of the dataset used in this study.

Dataset characteristic	Value
Total number of referrals	12,339
Number of unique LSOAs	3936
Number of columns (variables)	36
Overlapping columns	5 columns—3 columns provided duplicate information on referral reason, requiring consolidation
Missing data, *n* (%):	0 (0.0) on all variables
Most common referral reason, *n* (%)	Cataract = 2123 (17.2)
Sex	
Male, *n* (%)	5346 (43.3)
Female, *n* (%)	6993 (56.7)
Age group	
0–17 years	952 (7.7%)
18–39 years	1148 (9.3%)
40–59 years	2171 (17.6%)
60–79 years	5621 (45.6%)
≥80 years	2447 (19.8%)

The table summarises the overall number of referrals, unique geographical units (Lower Layer Super Output Areas; LSOAs) and dataset variables. It also reports data completeness, the most common reason for referral and the demographic distribution of patients by sex and age group.

**Fig. 1:**
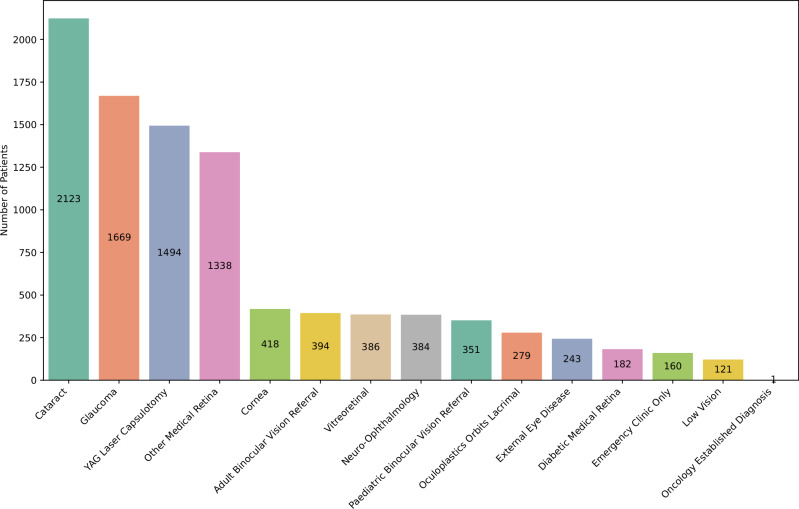
Distribution of optometry referrals by ophthalmology secondary care service. Bars represent the number of referrals per service type (e.g., cataract, glaucoma, cornea), reflecting the provisional diagnoses recorded by referring optometrists. The figure illustrates the relative burden of different referral categories within the dataset.

Within the dataset, three levels of referral priority were identified: routine referrals were the most frequent, totalling 11,130 (90.2%), followed by urgent referrals at 1049 (8.5%) and emergency referrals comprising 165 (1.3%). However, it is important to note that the dataset reflects only referrals captured within the electronic system. Emergency referrals made directly to Accident and Emergency (A&E)—outside the digital infrastructure—would not be represented. Figure 1 shows the number of referrals by eye condition. Cataract was the most common condition referred, with 2123 (17.2%) referrals, followed by glaucoma with 1669 (13.5%) referrals and referrals for YAG laser capsulotomy with 1494 (12.1%) referrals.

Interestingly, ~14% of referrals were directed within primary eyecare (i.e., optometry to GP or inter-optometry referrals) rather than to secondary care ophthalmology. Of these, 88% were sent to GPs. Inter-optometrist referrals can arise within enhanced eye care pathways, designed to reduce the number of referrals to ophthalmology in secondary care services. Examples include the Glaucoma Enhanced Referral Scheme (GERS), which accounted for 8.1% (142/ 1744) of inter-optometrist referrals within the primary eyecare and the Enhanced Cataract Referral Service programme, accounting for 1.7% (30/ 1744).

### Regression Analysis (Aim 2)

**Fig. 2:**
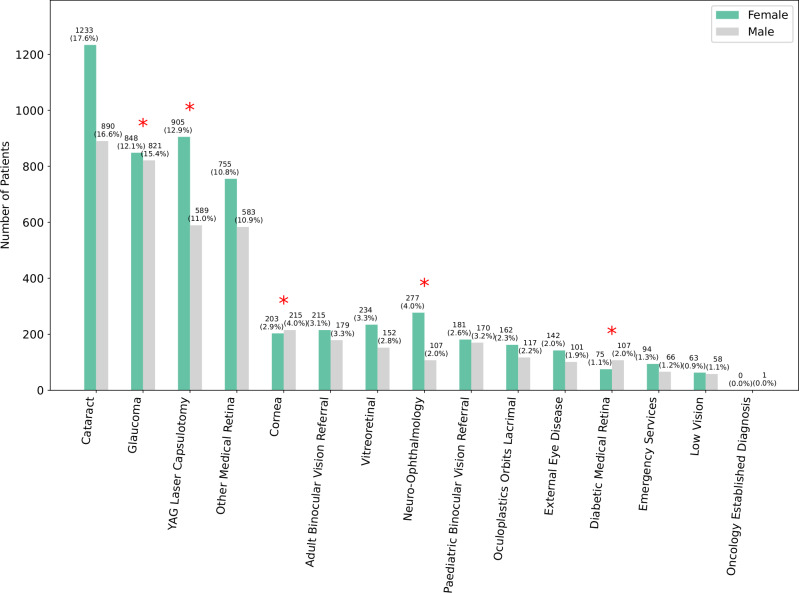
Distribution of optometry referrals by patient sex across ophthalmology secondary care services. Bars display both absolute referral counts and proportional percentages for males and females. Categories marked with an asterisk (*) indicate a statistically significant difference in the proportion of referrals between sexes, highlighting areas where referral patterns differ by gender.

The analyses, illustrated in Fig. 2 shows the proportions of males and females referred for each condition studied. Following Bonferroni correction (which established a modified threshold for statistical significance of *p* = 0.002), differences for the sexes were determined to be statistically significant (for neuro-ophthalmology, cornea, glaucoma, diabetic medical retina and YAG laser capsulotomy). For example, the proportion of referrals for YAG laser capsulotomy was higher in females (12.9%) than in males (11.0%). Although the absolute difference was only 1.9 percentage points, this difference was statistically significant (*χ*² = 11.99, *p* = 0.0005), likely due to the large sample size (*n* = 12,339) and consistent trend across referrals.

**Fig. 3:**
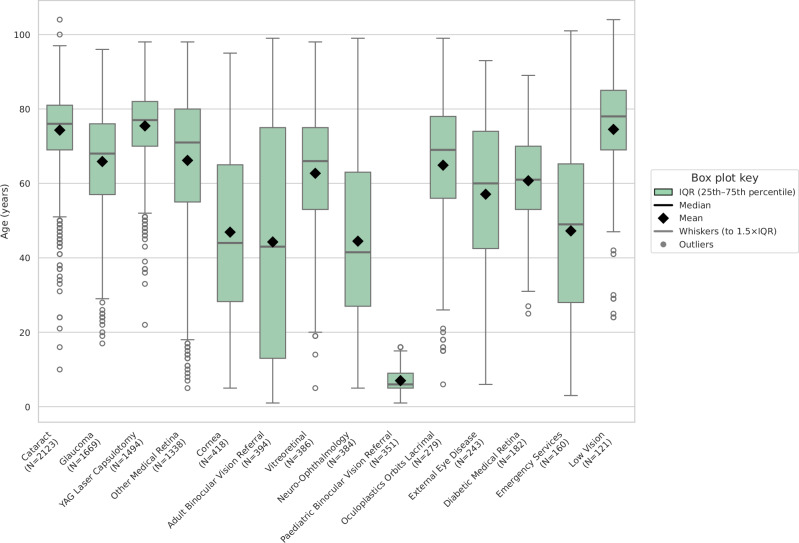
Age distribution of referrals to ophthalmology secondary care services. Box plots show patient age by referral category, indicating the median, mean (diamond), interquartile range (IQR) and outliers. Most referrals for cataract, YAG laser capsulotomy, glaucoma and medical retina services involved older adults, while corneal and binocular-vision referrals were notably younger. Only categories with ≥20 referrals are shown. Excluded: Oncology Established Diagnosis (*N =* 1).

In the overall cohort, the age profile was, as expected, skewed towards older adults: nearly half of participants were aged 60–79 years (45.6%) and a further fifth were ≥80 years of age (19.8%), with only 7.7% < 18 years of age (Fig. 3). Within secondary eyecare, referrals for cataract and YAG laser capsulotomy were overwhelmingly from older adults (cataract: 92.5% ≥60 years; YAG laser capsulotomy: 93.6% ≥ 60 years). Glaucoma referrals also showed an older age distribution (70.8% ≥60 years), although a substantial minority were aged 40–59 years (24.9%) (Fig. 3). Medical-retina referrals followed a broadly similar pattern (Other Medical Retina: 69.9% ≥ 60 years; Vitreoretinal: 66.0% ≥ 60 years), with smaller proportions in the younger age bands. In contrast, corneal referrals were markedly younger (41.9% < 40 years and only 32.5% ≥ 60 years), reflecting the mixed aetiology typically observed in corneal disease.

### Spatial Distribution of Referrals and Socioeconomic Deprivation (Aim 3)

This part of the dataset analysis found no issues when linking part-postcodes to LSOAs. Although a common challenge in such linkage processes is that not all postcodes correspond accurately to LSOAs—resulting in unmatched entries or requiring manual matching—this was not the case in this analysis.

**Fig. 4:**
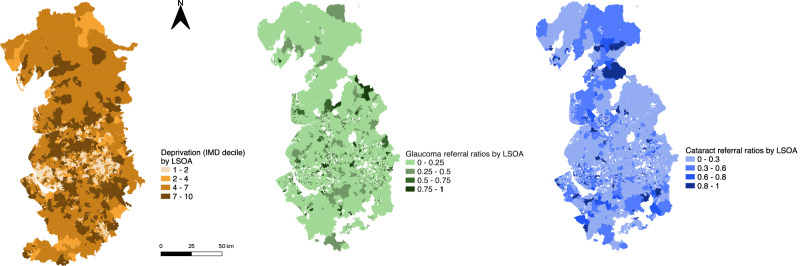
Three spatial maps showing deprivation and referral patterns across the convenience sample. Left: Index of Multiple Deprivation (IMD) decile for each Lower Layer Super Output Areas (LSOA) (1 = most deprived, 10 = least deprived). Centre: glaucoma referral ratio by LSOA. Right: cataract referral ratio by LSOA. Together, these allow visual comparison between referral patterns and area-level deprivation.

Spatial distributions of deprivation and referral ratios are illustrated in Fig. 4. The maps show deprivation by LSOA across the Northwest of England alongside the spatial patterns of cataract and glaucoma referral ratios. Analysis revealed a statistically significant but very weak positive correlation between deprivation and cataract referral ratios (*r* = 0.07, *p* < 0.001, 95% CI [0.04, 0.10]), suggesting slightly higher referral rates in less deprived areas, although the effect size was minimal and unlikely to be of practical significance. In contrast, no significant association was found between deprivation and glaucoma referral ratios (*r* = 0.007, *p* = 0.69, 95% CI [–0.03, 0.04]), indicating that glaucoma referrals were broadly uniform across deprivation levels.

## Discussion

This study appears to be the first to test the feasibility of evaluating a large dataset of electronic primary care optometry referrals in England. The analysis examined the suitability of electronic optometry referral data for three analytical approaches: descriptive, logistic regression and spatial analysis. A total of 12,339 optometry referrals were analysed. While this study likely represents the largest evaluation of optometry referral datasets to date, it serves as a feasibility study to inform future research, which should include a larger and more representative dataset collected over an extended timeframe. Importantly, the present dataset was restricted to referrals made within a single month (November 2023) and confined to the Northwest of England. These constraints limit the generalisability of the findings beyond this specific period and region. A further limitation is that the dataset only includes referrals transmitted via the electronic referral platform from primary care optometrists. While this route is expected to comprise most optometry referrals, it does not capture patients who present directly to hospital eye services or Accident and Emergency departments, nor actively managed via alternative pathways such as walk-in urgent eyecare clinics, traditional paper-based referrals, or private sector services. While this feasibility study focused on the available referral dataset, we acknowledge that assessing referral appropriateness and accuracy is an important area of future research. With the increasing use of large-scale electronic records in secondary care, future studies could link referrals with clinical outcomes to evaluate appropriateness and accuracy more robustly. Such work will also require effective data sharing and the development of collaborative interfaces between primary and secondary eyecare services. Nevertheless, the results provide an important foundation for future investigations into the nature of optometry referrals and associated eye conditions.

Categorising referral types presented the main challenge during the analysis. For example, “Ophthal not specified” could not be categorised by precise referral conditions. All the referral types were related to eye conditions, and some were requests for services from within primary eye care. Many eye conditions were labelled with the same name but differed in spelling, punctuation, or even capitalisation. As a result, the initial dataset included more than 50 referral types.

The results showed that descriptive analysis could successfully organise optometric referral data providing information about patients’ demographic details, reasons for referrals, urgency of referral, types of eye conditions referred and the total number of referrals in each NHS CCG. Descriptive analysis was selected as the initial method to establish a baseline for exploring optometry referral patterns. One of the benefits of this aim was the ability to explore the different types of eye conditions being referred, an investigation that has previously relied upon paper-based methods. The evaluation revealed that some conditions dominate the referral process. Cataracts were the most commonly referred condition, accounting for 17.2% of all referrals, followed by glaucoma at 13.5%. Although the sample in this feasibility study may not be representative of the UK as a whole, these findings do align with previous studies on the dominant eye conditions being referred, although not the percentages, such as Evans et al. [13], who reported 28% of referrals for cataracts and 25% for glaucoma. Similarly, Davey et al. [27] found that lens disorders, including cataracts, posterior capsular opacification and lens subluxation, accounted for 27% of referrals, while glaucoma comprised 20%.

Different schemes have been introduced to decrease the number of glaucoma referrals in England, with an enhanced General Ophthalmic Services being evident elsewhere in the UK to achieve the same effect. Additionally, in some areas, all glaucoma referrals must first pass through an enhanced community service. Henson et al. [8] reported that a community refinement scheme reduced glaucoma suspect referrals by 40%. More recently, Gunn et al. [28] found that the Manchester Glaucoma Enhanced Referral Scheme (GERS) reduced false positive referrals by 15.5%. In the current study, GERS and Glaucoma Repeat Reading (GRR) schemes received a small percentage of referrals, accounting for ~8% and ~1.5% of total referrals to a primary care practitioner, respectively.

Primary datasets can be used to explore the relationship between patients’ sex and referral patterns. The analysis method for the second aim indicates multiple significant associations between eye conditions and sex, as shown in Fig. 2. However, these differences may not reflect inherent sex-based predispositions for conditions such as cataract or sub-categories of glaucoma, which may show limited or no sex-specific prevalence differences in the general population. Instead, broader demographic and behavioural factors likely influence referral patterns. For instance, women typically live longer and are more likely to engage with primary care services, including routine eye examinations [29–31]. These factors may increase the likelihood that females comprise a larger proportion of patients referred for further ophthalmic evaluation. Such contextual analysis enhances understanding of how demographic dynamics—not just disease biology—shape clinical pathways [30].

From a feasibility perspective, the multiple regression models were successfully fitted to the dataset of 12,339 referrals, with minimal computational demands. The absence of missing data eliminated the need for imputation or exclusion strategies, ensuring analytic integrity. The robust sample size likely conferred adequate statistical power to detect differences where they existed, particularly for common referral categories. Statistical significance in some associations is likely to have been driven by the large sample size, even when the effect sizes were relatively small. As such, future findings on representative samples should be interpreted with confidence in the capacity of datasets to reveal meaningful effect sizes across the examined variables.

The visualisation maps produced in this study represent a potential advancement in understanding the geographical distribution of referrals. By linking referral data to the IMD, the maps may provide a detailed view of referral patterns relative to deprivation levels. To our knowledge, this is the first study to produce maps of optometry referrals at such a large scale. Such maps could be produced to highlight eye condition hotspots and trends over time, potentially facilitating exploration of eye care health promotion and care pathway developments. One advantage here is that these data permitted patient partial postcodes and referrals data to be explored, overcoming challenges noted in studies such as Harper et al. [32], where patients could choose sight test locations unrelated to their residential areas and where these authors were exploring deprivation set against postcodes for optometry provision versus patients’ residences.

Additionally, the scarcity of large datasets on optometry referrals or difficulties in accessing such data has limited prior research. The current findings show how this gap may be bridged, offering potential future insights into the relationships between deprivation, referral patterns and eye condition proportions, as explored by Wong et al. [33] and Woodward et al. [34].

## Conclusion

This feasibility study demonstrates that the statistical and analytical methods employed here are both practical and robust for the evaluation of optometric referrals. Importantly, these approaches are scalable and adaptable, supporting their application to larger, more diverse and representative datasets. Such extension could facilitate broader exploratory analyses, enabling the identification of systemic patterns, service utilisation disparities and temporal trends in referral behaviours. This approach has the potential to influence referral policy and technology infrastructure, and could offer opportunities to explore optometrists’ levels of training and accreditation, as well as referral quality and quantity. In this way, the feasibility findings from the current research lay the groundwork for more in-depth investigations. The next phase of the current research project will involve analysing a dataset encompassing referrals from across Greater Manchester over a 2-year period; an evaluation anticipated to offer a more representative basis for assessing referrals, which in turn will offer an opportunity to explore the changing dynamics of optometry referrals in the future, aiding the planning of equitable access to care pathways within primary and ophthalmology in secondary care services.

## Data Availability

The dataset used in this study was provided by the North West Electronic Eyecare Referral System (EeRS) team. Access to the data is restricted due to governance and confidentiality agreements. Further information regarding data access may be available upon reasonable request to the EeRS team, subject to approval.
